# A prospective investigation of direct and indirect home care activities in three rural Norwegian municipalities

**DOI:** 10.1186/s12913-018-3794-2

**Published:** 2018-12-18

**Authors:** Berit Irene Helgheim, Birgithe E. Sandbaek, Line Slyngstad

**Affiliations:** Molde University College, Specialized University in Logistics, Molde, Norway

**Keywords:** Prospective study, Activity data, Rural home care services, Direct and indirect home care, Process investigation

## Abstract

**Background:**

Home care providers struggle to manage their day-to-day work, which is increasing in volume and complexity. In general, they are expected to achieve more with the same planning methods, resources, and capacity. To meet emerging needs and use the available resources more effectively and efficiently, evidence and strategies are needed to inform planning methods for home care services. However, limited data are available to inform this change. This paper investigated the amount of time used to carry out direct activities and six indirect activities across three rural Norwegian municipalities (M1, M2 and M3).

**Methods:**

Home care staff recorded data over 8 weeks in 2016; the majority of the staff used a smartphone application and some staff used a manual form to report the durations of the activities.

**Results:**

The median time spent on direct activities was 11–13 min, and this work constituted less than 50% of the total work in the three municipalities. The median driving time was 5–7 min, which accounted for 43–54% of the total indirect work. Administration, particularly reporting and documentation, displayed greater differences across the municipalities, together accounting for 38–50% of the total indirect time. M2 and M3 used substantially more time for documentation, including 20 min in M2 and M3, in contrast to only 1 min in M1. Similarly, the median reporting times were 30 min (M2) and 28 min (M3), compared with only 17 min in M1.

**Conclusions:**

Home care staff spent less time on direct activities than on indirect activities, of which several activities have the potential for change. These results may help managers utilize resources effectively and plan appropriately, and they may also serve as a basis for future research to identify areas with improvement opportunities and, in turn, make more time available for direct patient care.

## Background

In recent years, Norwegian home care services have witnessed a substantial growth in demand, which largely reflects the aging population and a shift from acute hospital services to primary care. Norwegian home care services are primarily free (publicly funded) and a municipal responsibility. Following the Coordination Reform approved in 2009 and enacted in 2012 [1], many tasks formerly completed by hospitals were allocated to municipalities, which were given extended responsibility for patients. The municipalities must ensure that all inhabitants receive the necessary health services, regardless of age and diagnosis [2] which also indicates that they are unable to limit admissions to their services. Administrative and documentation requirements within home care services have also increased during this period, which has resulted in less time for patient care. Changing patient demographic characteristics and earlier hospital discharge have increased both the number of patient referrals and the complexity of these patients’ health needs; these changes have resulted in very complicated planning service demands in home care. Rural areas in western Norway with a great deal of land and a widespread population face additional challenges in terms of the travel time, winter climate (e.g., long periods with snow and ice), access to additional staff members (e.g., in case of sick leave), and after-hours availability of nearby general practitioners.

Currently, the existing home care workforce is struggling to manage their day-to-day work, which staff members often perceive to be highly unpredictable. Charged with additional work assignments with increasing complexity and great variability in daily volumes, staff members at the point of care are expected to achieve more with the same resources, planning methods, and capacity.

The workload performed in health care services comprises a broad and complex set of activities [3]. The process involves all of the activities, which, together and in a certain order, constitute the home care provided to a patient. These activities may be grouped into direct and indirect care. Direct care, also referred to as “face-time,” is the time spent with a patient. Indirect care consists of both indirect patient activities and nonpatient-related activities. In this paper, we do not distinguish between the latter two types of activities. There may also be dependency between activities, which indicates that one activity must be performed before another activity. Some dependencies are obvious and static, such as driving before and after providing direct patient care; however, other dependencies can be more random. For example, one indirect activity is to consult the physician before the caregiver drives to the patient. In other situations, the dependency could work in the opposite direction. The randomness of these dependencies makes planning even more challenging, and it is even more important to have an estimate of the expected workload for the activities.

According to Cooper [4] there is a lack of published literature on the workload in rural home care services. However, researchers have provided insights into some of the activities involved. The activity of driving is one of the major research fields related to home care services. Researchers have reported a decrease in driving time and a general increase in efficiency with the use of an optimization algorithm [5–7] and have developed systems to eliminate manual staff scheduling and route planning. These authors have reported increases in efficiency of 10–15% and a 29% decrease in driving time [6]. Another study used optimization to minimize travel distance [5].

However, in many Norwegian municipalities, existing tools for optimizing driving and staff scheduling are not compatible with the municipality’s patient administration and documentation system. Therefore, the overall planning of home care services is often ad hoc/experienced-based and performed manually, a time-consuming process that likely results in a suboptimal outcome.

Thomas and Davies [8] studied hospital activities, dividing the work into seven groups: patient treatment, patient care, administration, communication, off-ward, housekeeping, and other. The assessment period was seven days. However, the nature of the services provided at a hospital differ from that of home care services.

Holm and Angelsen [9] measured three home care activities, including driving, visiting time (indirectly including documentation), and other, using GPS data from the home care staff members’ cars for one week. In contrast to these authors, we use prospectively recorded data during 8 weeks and examine seven different activities. If managers want to change and release time from more indirect work to face time, they must have an overview of the time spent on different activities. In Holm and Angelsen [9] research, most indirect activities are indirectly captured in the three activities they investigate, whereas we recorded these activities separately.

The use of electronic devices and health information systems in home care service has changed a lot the last decade. Researchers have explored and reported clinical/medical benefits of home care technology in general [10]. Although use of electronic information systems in primary health services is rapidly developing, there is still variation between different countries. In many developed countries, such as Scandinavia, the Netherlands and the UK, use of electronic system has been more or less universal for several years, whereas adoption rates has been slower in both Canada and the United States [11]. Many information technology systems have integrated solutions for mobile documentation. However, there is little information on the extent to which these mobile device solutions have been adopted. In Norway, this is an economical issue for the municipalities. Currently, research in this area is largely lacking.

With increasing demands for home care services, it is essential to ensure sufficient time for direct patient care. Without additional resources, this can be achieved, for example, by allocating time from indirect activities. To identify areas eligible for change, information on how time is spent on various home care activities becomes essential. Currently, there is limited information on how time is spent on various home care activities.

The objective of this paper is to investigate the time spent on direct activities and six indirect activities in three rural municipalities. We use prospectively recorded data recorded by home care staff using a smart phone application or manual recording. The paper analyzes the distribution and proportion of time spent on these activities at a detailed level. Our results may be used to improve existing planning and scheduling methods to achieve more effective and efficient utilization of available resources, and they may also serve as a basis for future research to identify new areas with improvement opportunities and, in turn, make more time available for direct patient care.

## Methods

The analyses were performed on activity data recorded by home care staff members in three rural municipalities (M1, M2, and M3) located in western Norway. The municipalities are similar in terms of the climate, geography, landscape, and distance from the municipality center to the nearest hospital; however, they differ regarding other characteristics. Selected characteristics, as well as planned and participating staff with the corresponding distributions of the staff categories in the three municipalities, are displayed in Table 1.

**Table 1:** Selected characteristics and staffing of the three municipalities

Variables	Municipality		
	M1	M2	M3
Characteristics			
Inhabitants (n)	9787	7445	6708
Land area (km2)	370	1502	352
Patients (n)	230	290	275
Planned staff			
Staffing per week (n)	104	165	150
Registered nurse (%)	53	52	40
Assistant nurse (%)	45	48	60
Assistant health care worker	2	0	0
Sum	100	100	100
Participating staff			
Average weekly staffing (n)	98	75	96
Registered nurse (%)	43	45	30
Assistant nurse (%)	37	34	57
Assistant health care worker	20	21	13
Sum	100	100	100

The total number of planned staff per week was 104 in M1, 165 in M2 and 150 in M3. The distribution of the planned staff categories varies in the three municipalities. Registered nurses (RNs) account for over 50% of the planned staff in M1 and M2 and only 40% in M3, where assistant nurses (ANs) are the largest planned category. Only M1 plans to use assistant health care workers (AHWs).

In general, there are only minor differences between the number of planned staff and the actual number; however, the actual distribution of the staff categories often differs from the planned distribution. For example, in cases of sick leave or on busy days, staff work overtime or temporary staff is hired. Due to poor access particularly to RNs but also to ANs, temporary workers are often assistant healthcare workers employed in part-time positions.

The average number of participating staff per week varied in the municipalities. In M1, the average number of staff participating per week was 98, while in M2 and M3, the number was 75 and 96, respectively. These numbers correspond to 94% (M1), 45% (M2) and 64% (M3) of the weekly planned staff (the percentages are not shown in the table).

In accordance with the planned categories, RNs accounted for the largest participating staff category in M1 and M2, while ANs were the largest participating staff category in M3. In all three municipalities, the proportions of assistant health care workers were far greater than the planned staffing, which most likely reflects the use of temporary workers.

### Data

A total of 46,750 observations were recorded over 8 weeks, from January 13 to March 8, 2016. The data included the types of activities, as well as the date and start and end times for each activity. Prior to the analyses, 706 (1.5%) observations were deleted because of coding errors, which resulted in 46,044 observations eligible for analysis. Coding errors were mainly caused by activities running more than 24 h because the user had not indicated the end of an activity.

### Activities

Direct care refers to the time the staff members spent with patients in the patients’ homes. Indirect care consists of 17 different activities and refers to activities in which the patient does not participate. Appendix provides a complete list of all activities. For analytical purposes, indirect activities were merged into six groups. Table 2 presents an overview of the main groups of activities and the number of observations of each activity by municipality.

**Table 2:** Direct and indirect activities

Group	Activity	M1	M2	M3	Total
Direct care	Direct	5110	5859	6728	17,697
Indirect care	Drive	5935	6111	7000	19,046
	Adm	1107	999	1341	3447
	Report	659	578	1202	2439
	Teach	20	20	44	84
	Docu	1680	402	692	2774
	Drug	179	127	251	557
	Total	14,690	14,096	17,258	46,044

i) “Drive” refers to driving or walking to/from the patients, including driving associated with food deliveries to the patients; (ii) “Adm” refers to administration, such as staff meetings, communication with other health providers, designing care plans, case management, tasks that involve other departments (e.g., the lab), and planning and support activities; (iii) “Report” refers to oral reports made between shifts; (iv) “Docu” refers to documentation, such as writing individual reports for a patient after each visit or other incidents; (v) “Drug” refers to preparing dispensers with medication for individual patients; and (vi) “Teach” refers to education, including teaching and training at the department level and external courses

### The workload measurement tool

To record their activities, the staff members used a time tracker application (app) installed on their smartphones or a manual form. Before the project started, there were three meetings with the staff in each municipality. At the first meeting, the staff was provided with information regarding the project. At the second meeting, the staff and researchers worked together to select, define and categorize the specific activities that would be recorded. At the third meeting, the staff was shown how to install the app on their smartphones and how to record and test the app. In the app’s menu, the most frequent activities appeared on the top, and only a certain number of activities could be shown at the same time, depending on the specific type of smartphone employed. As none of the smartphones were able to display all activities in one window, the members of the staff needed to scroll down to find less frequent activities. They logged onto the app using an anonymous key and monitored their work by selecting an activity and then pressing the start/stop button at the beginning/end of each activity. After the completion of the study period, the app provider transferred an excel file that contained the registrations to the authors. All costs for internet use were reimbursed by the municipalities.

Some of the staff members recorded the activities manually because they did not have access to a smartphone or they preferred to use a manual registration form for other reasons. The manual registration form contained the same activities as the app. The staff noted the date and start and end times for each activity. After the registration period, the data were manually transferred to excel by hired workers who were independent from the study. Table 3 displays the number of different registration types for each municipality. The number of manual recordings was greatest in M2 and M3; however, there was no significant difference in the distribution of the times for data collected electronically and manually. In M1, only 3% of direct work and 2% of indirect work were recorded manually. Both of these displayed a minor but significantly different time distribution for data recorded manually and electronically.

**Table 3:** Number of observations and time distribution of electronically and manually recorded data

Municipality	Activity	Observations			Duration (minutes)				p
		Total	Electronic	Manual	Electronic		Manual		
		n	% of total	% of total	p50	(IQR)	p50	(IQR)	
M1	Direct	5110	97	3	11	(6–23)	8	(5–11)	0.00
	Indirect	9580	98	2	6	(2–16)	7	(1–11)	0.01
M2	Direct	5859	13	87	12	(8–20)	14	(8–23)	0.10
	Indirect	8237	14	86	8	(4–19)	7	(4–16)	0.36
M3	Direct	6728	80	20	12	(6–25)	11	(7–21)	0.30
	Indirect	10530	17	83	8	(3–18)	7	(3–15)	0.12

The data were analyzed using Stata version 15.1. The Mann–Whitney test was used to calculate the statistical significance of the observed differences in the duration of the different activities by municipality and record mode (electronic or manual). For statistical analyses, *p* < 0.01 was considered statistically significant.

## Results

The total work during the 8 weeks examined amounted to 3916, 3869, and 4849 h in M1, M2, and M3, respectively. The corresponding numbers of observations were 14,690, 14,096, and 17,258, respectively (Tables 2 and 4).

**Table 4:** Total time spent on direct and indirect patient care

Activity	Municipality	Duration (minutes)				Duration (hours)
		median	IQR	mean	total	%
Direct	M1	11	(6–23)	20	1710	44
	M2	13	(8–22)	19	1878	49
	M3	12	(6–24)	21	2391	49
Indirect	M1	6	(2–16)	14	2206	56
	M2	8	(4–17)	15	1991	51
	M3	8	(3–17)	14	2458	51
Total	M1	8	(3–18)	16	3916	100
	M2	10	(5–20)	16	3869	100
	M3	10	(4–20)	17	4849	100

### Direct activities

There were only minor differences in the time distributions for the direct activities among the municipalities (Table 4). More specifically, the median for the direct work time for M1 was 11 min, compared with 13 min for M2 and 12 min for M3. Direct activities constituted 44% of the total work in M1 and 49% of the total work in M2 and M3.

### Indirect activities

The median work time for the indirect activities was 6 min in M1, and 8 min in M2 and M3 (Table 4). Table 5 displays how time is spent on the indirect activity subgroups. Apart from M1 with a low documentation time, driving, administration, reporting and documentation were the most time-consuming indirect activities in terms of work hours.

**Table 5:** Time spent on indirect activities

Activity	Muni.	No Obs	Median (min.)	(IQR) (min.)	Mean (min.)	Sum dur. (hrs)	Indir.	Indir of tot hrs^a^
Drive	M1	5935	7	(3–15)	12	1183	54%	30%
	M2	6111	5	(3–11)	10	979	49%	25%
	M3	7000	6	(3–12)	9	1065	43%	22%
Adm	M1	1107	11	(3–33)	26	487	22%	12%
	M2	999	10	(4–26)	23	386	19%	10%
	M3	1341	7	(2–20)	17	376	15%	8%
Report	M1	659	17	(9–27)	23	255	12%	7%
	M2	578	30	(20–45)	32	313	16%	8%
	M3	1202	28	(15–37)	28	569	23%	12%
Docu	M1	1680	1	(0.5–2)	4	99	4%	3%
	M2	402	20	(15–30)	26	173	9%	4%
	M3	692	20	(11–34)	25	287	12%	6%
Drug	M1	179	21	(7–57)	46	137	6%	4%
	M2	127	40	(15–75)	53	113	6%	3%
	M3	251	17	(7–48)	33	138	6%	3%
Teach	M1	20	70	(9–219)	136	45	2%	1%
	M2	20	35	(24–68)	80	27	1%	1%
	M3	44	10	(3–43)	32	23	1%	0%
Total indirect	M1	9580	–	–	–	2206	100%	56%
	M2	8237	–	–	–	1991	100%	51%
	M3	10,530	–	–	–	2458	100%	51%

^a^ Calculated based on the total number of hours provided in Table 4

The median driving time ranged from 5 to 7 min in the three municipalities. Driving constituted the largest proportions of indirect work time at 54, 49 and 43% in M1, M2 and M3, respectively.

The median time spent on administration was 11 min in M1 and 10 min in M2, while it was only 7 min in M3. Administration was the second most time-consuming indirect activity in both M1 (22%) and M2 (19%), whereas it only accounted for 15% of all time spent on indirect activities in M3.

The median time spent on reporting was 17 min in M1, 30 min in M2 and 28 min in M3. Reporting represented the third most time-consuming indirect activity in M3 (23%); however, it only accounted for 12% of the total time spent on indirect activities in M1 and 16% in M2.

The median documentation time was 20 min in both M2 and M3, which was extensively longer than the corresponding time of 1 min for M1. Extrapolated to hours, this finding indicates that over the course of the 8 weeks, substantially more time was spent on documentation in M2 and M3 than in M1. Documentation comprised 4, 9, and 12% of the total indirect time in M1, M2, and M3, respectively.

The median duration spent on medication was 21 min in M1 and 17 min in M3, both of which were substantially longer than the median of 40 min in M2. Medication accounted for 6% of the total indirect time in all three municipalities.

The median duration for teaching was 70 min in M1, 35 min in M2, and 10 min in M3. This activity accounted for only 1–2% of indirect activities.

## Discussion

This paper uses prospectively recorded data and documents how time is spent in home care activities in three rural municipalities. The results demonstrated only minor differences across the municipalities in the time distribution for direct activities; however, for some indirect activities, the differences were greater. The median time spent on direct activities ranged from 11 to 13 min in the three municipalities. This activity accounted for 49% of the total work time in M2 and M3 and only 44% in M1. The results are somewhat different from the findings of a study conducted by the Norwegian Association of Local and Regional Authorities [12], in which the overall results showed that direct activities accounted for 30 to 46% of the total work time. However, in this previous study, all staff completed forms manually and only for 2 weeks. According to the respondents, many of the forms were completed at the end of the day or shift. Moreover, the study provides no information on the number of participants or the number of observations.

The six indirect activities did not include direct contact with the patients. Examining the median time spent on specific indirect activities, we observed large differences in some activities across the three municipalities. Driving was the most time-consuming indirect activity in all three municipalities, with relatively equal median durations in the three municipalities. However, as a proportion of the total time spent on indirect activities, driving accounted for 54, 49 and 43% in M1, M2 and M3, respectively. The corresponding proportions of driving versus total worktime were 30, 25 and 22%, respectively.

These proportions are greater than Holm and Angelsen’s [9] finding that driving accounted for 18–25% of the total time spent working. This may be explained by a difference in the settlement pattern across the three municipalities. For example, if M1 has a more dispersed settlement pattern compared with the areas studied in previous research, it will influence the time spent on driving. However, this difference from previous findings could also indicate that the municipality in our research had less optimal driving routes. Research on route planning in home care services has shown that improvements are possible with the use of tools to optimize the driving routes [13]. Another interesting point is that our findings do not reflect the land area or the number of patients in the municipalities. For example, despite having more patients and approximately four times the land area, M2 had almost the same driving time as M1, which indicates that the settlement pattern is more important than the land area for driving time.

There were substantial differences in the documentation time, with M1 deviating from M2 and M3. The median for M1 was only 1 min, whereas the medians for M2 and M3 were both approximately 20 min. The staff in M1 completed their documentation electronically in their cars after seeing each patient. They also had a “tick off” system, which indicates that if their work was completed as planned, they ticked off the task, without writing further notes. If there were changes, they added a short note; however, all documentation was completed immediately after seeing each patient. In contrast to M1, in M2 and M3, the staff had to take notes while they were with the patients and then complete the electronic registration at the end of their shifts. Thus, they had to complete this work twice, which may explain the substantial difference in the time spent on this activity.

M1 also deviated from M2 and M3 in terms of the time spent on reporting. M1 had a median of approximately 17 min spent on reporting, whereas M2 and M3 both had medians of approximately 30 min. Reporting is the oral description and explanation of what was done during the shift. This activity is accomplished in the overlap between shifts. There might be an association between the activities of documentation and reporting. The time in M1 was significantly lower than that in the other two municipalities for both of these activities, which may indicate that the structured documentation influences the oral reporting. This finding might imply that municipalities can save time by implementing a structured electronic documentation system. On a yearly basis, the savings potential for M2 and M3 on these two activities could be 1000 working hours. In the research literature on the effects of implementing information systems in health care, there is some support regarding time savings as a result of transferring from manual to electronic registration [14].

An interesting observation is that M1 had the lowest share of time used on direct activity but spent more time on administrative tasks. In informal conversations in this municipality, it was determined that the staff use a substantial amount of administrative time on the electronic system. This finding implies a transfer of time saved by using electronic documentation to administrative tasks. Previous research on implementing electronic systems in health care organizations has reported similar findings. For example, Takian et al. [15] reported perceptions of a new system being obstructive and inflexible, and Noblin et al. [16] found training to be important. These authors also highlighted that the training needed to be at an individual level. M1 had implemented the electronic registration system only 6 months prior to the research, which may explain why staff members in this municipality spent substantial time on the system. It is likely that this will decrease over time and that the time saved could be allocated to additional direct time.

For the activity of teaching, there were also major differences. The median time spent on teaching was 70 min in M1, 35 min in M2, and 10 min in M3. M1 had a more structured teaching system, including 1 h of teaching for the whole staff every other week in their shift schedule. In contrast, M2 and M3 more randomly organized their teaching. It is possible that the observation period was too short to accurately reflect this activity for M2 and M3. In the literature on home care services, many researchers have reported the effects of training on specific objectives [17]. Tourangeau et al. [18] have also highlighted the importance of training for providing quality client care. However, we did not identify other researchers reporting findings on or making recommendations regarding the time spent on teaching in home care services.

More time was spent on drug administration in M2. According to the nurses, the most consuming part of this activity was refilling medication dispensers for individual patients. The 75th percentile for the time spent on this activity was 75 min for M2, whereas it was 57 min and 48 min for M1 and M3, respectively. One explanation for this difference might be that M2 had a more complex case mix, which made the refill process more time-consuming. The experience and organization of the medical administration system might also differ and have an impact on the amount of time that was spent on this activity. The use of electronic automatic medicine dispensers may contribute to time savings for both drug administration and driving, particularly if many patients are delivered medication several times per day or week.

### Limitations

There were several limitations to this study. First, not all staff members participated. Thus, we do not have a full overview of the total time spent. However, the municipalities confirmed that the distribution of the participating staff categories did not substantially diverge from the actual distribution, which indicates that the percentages of the total work time are likely to be representative of the staff’s activities in each municipality.

With the large data size, we believe that this limitation will not have an impact on the median times. Second, home care service staff members often multitask. They might simultaneously take a phone call, discuss cases with other individuals, and carry out other activities. This type of activity was not recorded in this project. Third, the participants used two different data recording instruments. For the manual recording, typing errors when manually transferring the data to Excel may have occurred, and the manual recording might have been more time consuming than the electronic recording.

For both manual and electronic recording, the number of activities may be too many. Thus, the accuracy of the individual activities may be skewed. Our recommendation is to focus on the 5–10 most important activities.

Furthermore, 85% of the data for M2 were manually recorded and for these data we could not verify if these data was continuously recorded. Prior to the initiation of the data collection in the municipalities, we provided instructions on the importance of filling in the forms continuously throughout the work day. However, we could not verify how the manual forms were actually completed. Finally, this study focused on three rural municipalities on the west coast of Norway, which indicates that our results many not be generalized to urban municipalities.

Another issue for electronic recording is that the app employed did not have an offline service. In this project, this was not a problem, because there was internet coverage in all three municipalities. However, for users with poor internet coverage, we recommend using an application that has an offline solution with the ability to synchronize the recorded data when online.

## Conclusions

With increasing demands for home care services, it is essential to ensure sufficient time for direct patient care. Without additional resources, this can be achieved, for example, by allocating time from indirect activities. To identify areas eligible for improvement, information on how time is spent on various home care activities becomes essential. Currently, there is limited information on how time is spent on various home care activities, which may impede efficient and effective planning.

This study uses prospectively recorded data to document the time spent on direct and six indirect activities in each of the three studied municipalities. The results show that home care service staff spent less time on direct activities than on indirect activities, of which several activities have the potential for time savings. Driving was the most time-consuming of the examined indirect activities. A better scheduling and route planning system could be beneficial. There is also potential for improvement in the activities of documentation, administration, and reporting. We found that in the one included municipality with an electronic registration system, substantially less time was spent on reporting and documentation. Additional gains may be achieved by the use of electronic medical dispensers through time savings in both drug administration and driving.

In general, it is important for managers to more closely investigate activities to utilize resources effectively and plan appropriately. The results in this paper may serve as a basis for future research to identify new areas with improvement opportunities and, in turn, make more time available for direct patient care.

## Appendix

Table 6.

**Table 6:** Complete list of activities

Activities	Specification
Direct	Nursing, including alarms
Drive	Travel
	Food delivery
Admin	Administration
	Purchase/Order
	Patient meetings (nonspecified patient is not participating)
	Patient meetings (specified patient is not participating)
	Phone calls from patients, relatives, and healthcare staff in general
	Phone calls - home care staff, communication and coordination
	Various assignments from public authorities
	Other patient-related activities
	Pharmacy visits by staff
	Grocery shopping for patient
	Laboratory visits for patient
Report	Reporting
Teach	Teaching
Docu	Documentation
Drug	Multidose medication dispensing

## Abbreviations

Admin: Time spent on administrative tasks
AHW: Assistant healthcare workers
AN: Assistant nurse
Dir: Direct activities with patients, face time
Docu: Time spent on written documentation
Drive: Driving time from one point to the next point
Drug: Time spent on administrative preparation of medicine for individual patients
Indir: Indirect activities that do not directly involve patients
IQR: Interquartile range
M1-M3: Municipality 1–3
Report: Time spent on reporting activities
RN: Registered nurse
Teach: Time spent on teaching and training personal and students
